# Reliable and Adaptive Probabilistic Forecasting for Event-Driven Water-Quality Time Series Using a Gated Hybrid–Mixture Density Network

**DOI:** 10.3390/s25247560

**Published:** 2025-12-12

**Authors:** Nadir Ehmimed, Mohamed Yassin Chkouri, Abdellah Touhafi

**Affiliations:** 1 Information System and Software Engineering (SIGL) Laboratory, National School of Applied Sciences of Tetouan, Abdelmalek Essaadi University, Tetouan 93000, Morocco; mychkouri@uae.ac.ma; 2 Department of Engineering Sciences and Technology (INDI), Vrije Universiteit Brussel (VUB), 1050 Brussels, Belgium; abdellah.touhafi@vub.be

**Keywords:** probabilistic forecasting, time series, deep learning, Mixture Density Networks, LSTM, water quality, event prediction, heteroscedasticity, uncertainty quantification, calibration

## Abstract

Real-time, reliable forecasting of water quality (WQ) is a critical component of sustainable environmental management. A key challenge, however, is modeling time-varying uncertainty (heteroscedasticity), particularly during disruptive events like storms where predictability decreases dramatically. Standard probabilistic models often fail in these high-stakes scenarios, producing forecasts that are either too conservative during calm periods or dangerously overconfident during volatile events. This paper introduces the Gated Hybrid–Mixture Density Network (GH-MDN), an architecture explicitly designed for adaptive uncertainty estimation. Its core innovation is a dedicated gating network that learns to adaptively modulate the prediction interval width in response to a domain-relevant, event-precursor signal. We evaluate the GH-MDN on both synthetic and real-world WQ datasets using a rigorous cross-validation protocol. The results demonstrate that our gated model provides robust calibration and trustworthy adaptive coverage; specifically, it successfully widens prediction intervals to capture extreme events where standard benchmarks fail. We further show that common aggregate metrics such as CRPS can mask over-confident behavior during rare events, underscoring the need for evaluation approaches that prioritize calibration. This science-informed approach to modeling heteroscedasticity prioritizes reliable risk coverage over aggregate error minimization, marking a critical step towards the development of more trustworthy environmental forecasting systems.

## 1. Introduction

Effective management of water resources necessitates accurate and timely forecasting of water-quality (WQ) parameters. Such forecasts are vital for early warning systems, operational control of water treatment facilities, and proactive environmental protection strategies [1]. Traditional forecasting methods often provide point estimates, which, while useful, fail to capture the inherent uncertainty in predictions. This uncertainty arises from various sources, including model limitations, measurement errors, and the stochastic nature of environmental processes [2]. Probabilistic forecasts, which provide a full predictive distribution or prediction intervals (PIs) at a given confidence level, offer a more complete picture of potential future WQ states, enabling more informed and risk-aware decision-making [3].

A significant challenge in WQ time series forecasting is the presence of heteroscedasticity, where the variance of the forecast error is not constant over time [4]. This phenomenon is often event-driven; for instance, storm events can trigger rapid changes and increased variability in parameters like turbidity and conductivity due to surface runoff and sediment resuspension. Standard time series models assuming homoscedasticity (constant variance) struggle to adequately represent this dynamic uncertainty, leading to PIs that are either too wide during stable periods or too narrow during volatile events, thus undermining their reliability and sharpness.

Deep learning models, particularly Long Short-Term Memory (LSTM) networks, have shown considerable success in WQ point forecasting due to their ability to capture complex temporal dependencies [5]. However, extending these to reliable probabilistic forecasting under event-driven heteroscedasticity remains an active research area. Mixture Density Networks (MDNs) [6] offer a flexible framework for modeling complex conditional probability distributions by parameterizing a mixture of Gaussian (or other) distributions using neural network outputs.

To address this challenge, we introduce the Gated Hybrid–Mixture Density Network (GH-MDN). While labeled ‘GH_MDN_StackedLSTM’ in the figures to explicitly denote its architectural backbone, we refer to the model as ‘GH-MDN’ throughout the text for the sake of readability. This novel framework is explicitly designed to mirror the underlying physical processes of WQ dynamics, combining a deterministic baseline to capture primary trends, a stochastic residual model to account for complex uncertainty, and a targeted, learn-able gating mechanism. This gate, which is the core innovation, uses a single, domain-relevant signal to adaptively modulate predictive variance, allowing it to be more interpretable than standard attention mechanisms. This paper provides a comprehensive validation of this approach. Through a robust 5-fold cross-validation on both synthetic and real-world data, including crucial ablation studies, we demonstrate that our MDN-based architecture addresses the critical reliability flaws of simpler baselines. Rather than focusing solely on aggregate error metrics—which can sometimes reward models that are dangerously overconfident during calm periods—we highlight that the GH-MDN provides robust calibration and trustworthy adaptive coverage. Critically, we show that the gating mechanism leads to superior calibration and adaptive risk-coverage compared to its non-gated counterpart, successfully widening prediction intervals to capture extreme events where other models fail, thereby prioritizing safety and reliability. Our analysis further provides a nuanced examination of these adaptive behaviors. Finally, to contextualize our work, we benchmark the model against a state-of-the-art multi-task architecture, exploring the fundamental trade-offs between dedicated uncertainty modeling and holistic system representation.

A secondary contribution of this work is to highlight the limitations of CRPS when used as the sole optimization target in event-driven, safety-critical forecasting. Although CRPS is a valuable metric overall, we show that in environmental risk applications, it may favor sharp but over-confident predictions. Since operational systems prioritize reliability and calibration, we propose an architecture explicitly designed to meet these requirements.

The remainder of this paper is structured as follows: Section 2 reviews related work in WQ forecasting and probabilistic deep learning. Section 3 presents the methodology, detailing the problem formulation, the datasets utilized, the proposed GH-MDN framework, and the training procedure. Section 4 describes the experimental design, including the baseline and benchmark architectures, prediction interval calibration strategies, and evaluation metrics. Section 5 presents and discusses the results. Finally, Section 6 concludes the paper and outlines future research directions.

## 2. Related Work

Water-quality forecasting has evolved from traditional statistical models to sophisticated machine-learning and deep-learning approaches.

### 2.1. Traditional and Early Machine-Learning Methods

Traditional time series models like AutoRegressive Integrated Moving Average (ARIMA) have been widely applied but often struggle with the non-linear and non-stationary nature of WQ data [7]. Early machine-learning methods such as Support Vector Regression (SVR) [8], Random Forests (RFs) [9], and Artificial Neural Networks (ANNs) [10] offered improvements by capturing non-linear relationships but often lacked mechanisms for robust uncertainty quantification or handling complex temporal dynamics effectively [11].

### 2.2. Deep Learning for Water-Quality Point Forecasting

Recurrent Neural Networks (RNNs), particularly LSTMs [12] and Gated Recurrent Units (GRUs) [13], have become state-of-the-art for WQ point forecasting. Their ability to learn long-range dependencies makes them well suited for time series data [14]. Convolutional Neural Networks (CNNs) have also been used, sometimes in hybrid architectures with LSTMs (e.g., CNN-LSTM), to extract spatial or short-term temporal features [5].

### 2.3. Probabilistic Forecasting Methods

Recognizing the limitations of point forecasts, research has increasingly focused on probabilistic forecasting. Quantile Regression (QR) directly models specific quantiles of the predictive distribution. QR-LSTMs extend this to deep learning, allowing for the estimation of PIs without distributional assumptions [15]. Bayesian Neural Networks (BNNs) learn distributions over model weights. Monte Carlo (MC) Dropout [16] provides a scalable approximation to BNNs by performing inference with dropout enabled, yielding multiple samples to construct a predictive distribution. Deep Ensembles [17] train multiple neural networks with different initializations (and, potentially, data shuffles) and average their predictions (or combine their output distributions) to improve robustness and quantify uncertainty. Mixture Density Networks (MDNs) [6] are a powerful class of models where the neural network outputs the parameters (weights, means, variances) of a mixture model (typically Gaussian). This allows MDNs to represent arbitrary conditional probability distributions, making them highly flexible for capturing complex, multimodal, and heteroscedastic uncertainty patterns [18]. MDNs have been applied in various time-series domains, including WQ.

### 2.4. Modeling Heteroscedasticity

Addressing heteroscedasticity is crucial for reliable probabilistic forecasts. Some deep-learning models, like DeepAR [19], directly predict parameters of a chosen probability distribution (e.g., mean and variance of a Gaussian or Negative Binomial), inherently modeling time-varying uncertainty. Modeling residuals explicitly is another approach. If a base model captures the main trend, a separate model can predict the distribution of its errors, which often exhibit heteroscedasticity [20]. Our work builds upon this by modeling residuals with an MDN. Gating mechanisms have been used in various neural network architectures, such as in LSTMs themselves or in Mixture of Experts models [21,22], to control information flow or combine specialized subnetworks. The concept of using a gate to adaptively modify model behavior based on certain input conditions or learned states is relevant here.

The proposed use of an explicit gating network is conceptually related to, but distinct from, other advanced deep-learning techniques. For instance, Mixture of Experts (MoE) models [21] also use a gating network but typically to select or weight the outputs of several “expert” sub-models, each responsible for the full prediction. Our approach is more targeted, using the gate solely to modulate the variance parameter of a single residual distribution. Similarly, while attention mechanisms could potentially learn to up-weight the influence of an event-triggering variable, they do so implicitly as part of a complex learned weighting scheme across all features. Our framework makes the link between the trigger variable and the variance response explicit, which can improve interpretability—a critical goal for modern forecasting systems, as exemplified by architectures like the Temporal Fusion Transformer [23]—and is conceptually related to the gating mechanisms in state-of-the-art Mixture-of-Experts foundation models, which dynamically route inputs to specialized subnetworks [24]. Our approach, however, uses this gating concept not for computational routing but for the targeted and interpretable modulation of predictive uncertainty.

Our proposed GH-MDN framework is novel in its hybrid structure that combines a deterministic baseline LSTM, a residual MDN for flexible distributional modeling of errors, and an explicit, learnable gating network that uses an auxiliary event-triggering WQ variable to adaptively scale the variance of the MDN’s predictive distribution. This targeted variance modulation for event-driven heteroscedasticity in WQ time series distinguishes it from existing approaches.

## 3. Methodology

### 3.1. Problem Formulation

Let $y_{t}\in R$ be the value of a target water-quality parameter at time *t*, and $x_{t}\;\in \;R^{D}$ be a vector of *D* exogenous and/or lagged endogenous features at time *t*. Given a historical sequence of observations up to time *t*, $\left\{\left(y_{1},x_{1}\right),\ldots ,\left(y_{t},x_{t}\right)\right\}$, the objective is to forecast the probability distribution of the target variable at a future time step t+h, $P(y_{t+h}|y_{1},\ldots ,y_{t},x_{1},\ldots ,x_{t+h})$. In this study, we focus on one-step-ahead forecasting, so h=1. The probabilistic forecast aims to capture not only the expected value but also the uncertainty associated with the prediction, a task for which deep learning has become the state-of-the-art approach, as surveyed in recent literature [25].

### 3.2. Experimental Datasets and Specific Feature Selection

To rigorously evaluate the model, we utilize three datasets ranging from controlled synthetic environments to complex real-world river systems. Each dataset requires specific feature-engineering strategies to capture its unique dynamics.

#### 3.2.1. Primary Dataset: Synthetic Water-Quality Dataset

To rigorously evaluate the model’s ability to handle event-driven heteroscedasticity, this study utilizes a purpose-built synthetic dataset. The dataset, named synthetic_river_wq_hourly_deep_learning.csv, was generated to emulate the key characteristics of real-world hourly water-quality time series. It spans two years of records for five WQ parameters: Turbidity (NTU), pH, Temperature (°C), Dissolved Oxygen (DO, mg/L), and Conductivity (μS/cm) for a single conceptual site, “Station_A”.

A critical feature of the data-generation process was the explicit introduction of event-driven volatility, as modeling such time-varying uncertainty (heteroscedasticity) is a well-known challenge in time-series analysis [20]. The generation algorithm (detailed in Appendix A) simulates periodic storm events, during which the Turbidity and Conductivity parameters exhibit sharp spikes and significantly increased variance. This design creates a challenging and realistic testbed for evaluating a probabilistic model’s ability to adapt its uncertainty estimates to changing environmental conditions, a primary objective of this research.

##### Feature Engineering and Preprocessing

The raw time-series data was processed to create features suitable for the deep-learning models, following standard preprocessing steps—such as feature scaling and cyclical time encoding—that remain crucial even for state-of-the-art architectures like Transformers [26].

Feature Engineering: The input features for the models include:

- Lagged values of the target variable.
- Cyclical time features: The hour of the day and day of the week were transformed using a standard sine/cosine encoding to preserve their cyclical properties (e.g., ensuring hour 23 is adjacent to hour 0).
- Potential exogenous features such as Rainfall and Flow were included as model inputs if available in the dataset, alongside endogenous and time features, as specified by the include-exogenous command-line flag.

Data Scaling: All features, including the target variables, are scaled to a [0, 1] range using ‘MinMaxScaler’ from Scikit-learn. The scaler is fitted only on the training portion of the data for each cross-validation fold to prevent data leakage from validation or test sets.

Sequence Generation: The time-series data is transformed into sequences of a fixed length *L*. For a sequence length L=72 (as used in our primary experiments), each input sample $X_{i}$ consists of feature values over *L* consecutive time steps, $\left[x_{i-L+1},\ldots ,x_{i}\right]$, and the corresponding target $Y_{i}$ is $y_{i+1}$.

#### 3.2.2. Validation Dataset: VUB Smart Water Network

To bridge the gap between synthetic experiments and practical application, we utilize real-world data from the Smart Water-Monitoring System operated by Vrije Universiteit Brussel (Belgium), collected through a network of robotic sensors from November 2021 to December 2023. This dataset contains hourly measurements of key water-quality parameters, including a volatile Turbidity signal. We selected this specific dataset to validate the model’s stability in an operational context, ensuring that the theoretical gains observed in synthetic tests translate to actual sensor hardware subject to real-world noise.

Preprocessing and Features: The raw sensor data first underwent standard preprocessing, including outlier removal and missing-value imputation, to ensure data quality. For modeling, we used historical target values and cyclical time features to establish baseline performance on real sensor hardware.

#### 3.2.3. Benchmark Dataset: Kenyan River System

To rigorously assess generalizability across different hydrological regimes, we utilize a real-world dataset characterized by complex, non-linear dynamics. The data originates from an autonomous, in situ water-quality monitoring station deployed in a river system in Kenya. The station, identified by the unique IMEI 861513064190226, contains a sensor package that records key environmental variables. The final, processed dataset used for this benchmark consists of hourly measurements of four primary water-quality parameters: dissolved oxygen (DO), pH, temperature, and electric conductivity (EC).

The raw data collected by the sensor platform underwent a comprehensive cleaning and preprocessing pipeline to create the structured, analysis-ready dataset used in this work. This process is critical for handling common issues associated with remote environmental sensing, such as transmission gaps, sensor noise, and biofouling. The pipeline consisted of the following sequential steps:

1. Temporal Aggregation: The sensor initially recorded data at a higher frequency. To standardize the time-series and smooth transient noise, the data was down-sampled to a consistent hourly interval using the mean value of all readings within each hour.
2. Outlier Detection and Removal: A statistical method was employed to identify and remove anomalous spikes. This step is a critical pre-processing stage for ensuring data reliability, where machine-learning approaches have recently proven highly effective for identifying such outliers in sensor data [27]. A 24 h rolling window was applied, and any data point that deviated by more than five standard deviations from the rolling mean was flagged as an outlier and removed.
3. Missing Value Imputation: Gaps from outlier removal and data transmission failures were filled using time-weighted linear interpolation.
4. Feature Engineering: The raw timestamps were used to engineer cyclical temporal features—hour_of_day, day_of_week, and day_of_year—which are essential for capturing diurnal and weekly patterns.

**Figure 1:**
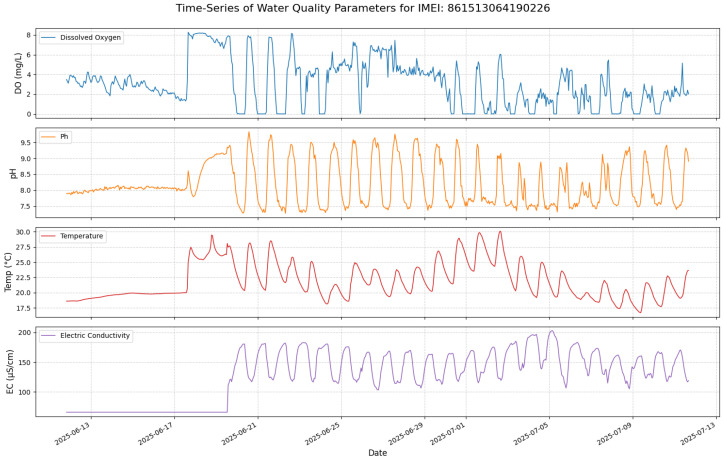
Time-series of the real-world benchmark dataset.

This multi-stage process resulted in a clean, hourly time-series dataset, which serves as the foundation for this benchmark analysis. A visualization of the processed time-series for the four target parameters is presented in Figure 1. The data exhibits strong non-stationarity, clear diurnal cycles, and abrupt, event-driven shifts that challenge standard forecasting models.

**Figure 2:**
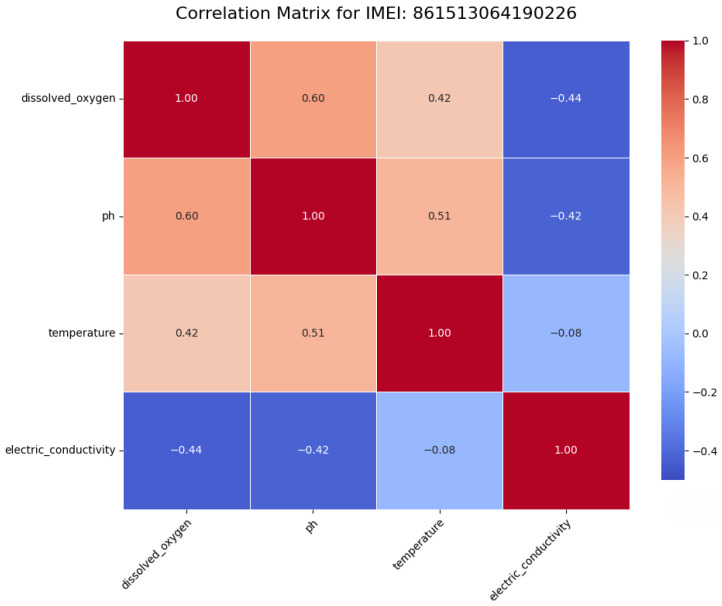
Static Pearson correlation matrix for the target variables at station ...90226.

An exploratory analysis was conducted on this processed data to understand the underlying dynamics. A static correlation matrix (Figure 2) reveals strong contemporaneous relationships, such as the positive correlation between pH and dissolved oxygen (0.60).

**Figure 3:**
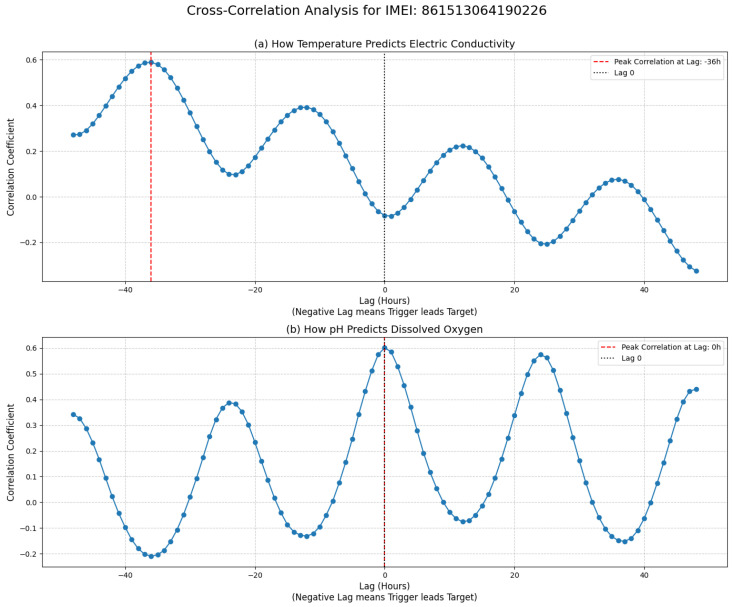
Cross-correlation analysis of benchmark variables.

To uncover the temporal dependencies essential for forecasting, a cross-correlation analysis was performed. This analysis reveals significant lead–lag relationships, providing a quantitative basis for assessing the potential of multivariate forecasting approaches. The key findings for station ...90226 are summarized in Table 1, where negative lags indicate that the trigger variable is a leading indicator for the target. For instance, temperature is a strong leading indicator for electric conductivity, with changes in temperature preceding changes in EC by 36 h (Figure 3a). Conversely, the relationship between pH and dissolved oxygen is almost perfectly in-phase (Figure 3b), indicating a tightly coupled diurnal process.

The characteristics revealed by this analysis—namely the non-stationary, event-prone nature of the time-series and the complex, time-displaced inter-parameter dynamics—motivate the selection of advanced probabilistic models for this benchmark. A suitable benchmark model must be capable of looking beyond univariate history and leveraging these rich, multivariate dependencies to produce accurate and reliable forecasts.

### 3.3. A Physically Inspired Modeling Approach

Standard deep-learning models often fail on WQ data because their monolithic architectures do not reflect the composite nature of the underlying system. Our proposed GH-MDN framework is explicitly designed to mirror these physical processes:

- The Baseline Process ($LSTM_{base}$): We hypothesize that the primary, predictable component of a WQ signal is driven by its recent history and smooth diurnal patterns. An LSTM is well suited to model this deterministic, autoregressive behavior.
- The Stochastic Residuals (${\mathrm{MDN}}_{res}$): We assume that the errors of the baseline model are not simple white noise but represent complex, unmodeled physical processes. An MDN is chosen to capture the potentially multimodal and heteroscedastic nature of this residual uncertainty, a state-of-the-art probabilistic approach that has been validated for quantifying uncertainty in water-quality indicators from complex observational data [28].
- The Event-Driven Volatility ($G_{event}$): We hypothesize that the largest source of heteroscedasticity is triggered by external hydrological events (e.g., storms), which are often signaled by a sentinel variable like Electrical Conductivity. A dedicated gating network is designed to explicitly learn this trigger–response relationship, directly modeling the system’s shift into a high-variance state.

This process-based decomposition is the core inductive bias of our framework, aligning our work with the growing paradigm of unifying machine learning and physical models for improved interpretability and performance in the geosciences [29].

### 3.4. The Proposed Gated Hybrid–Mixture Density Network (GH-MDN) Framework

**Figure 4:**
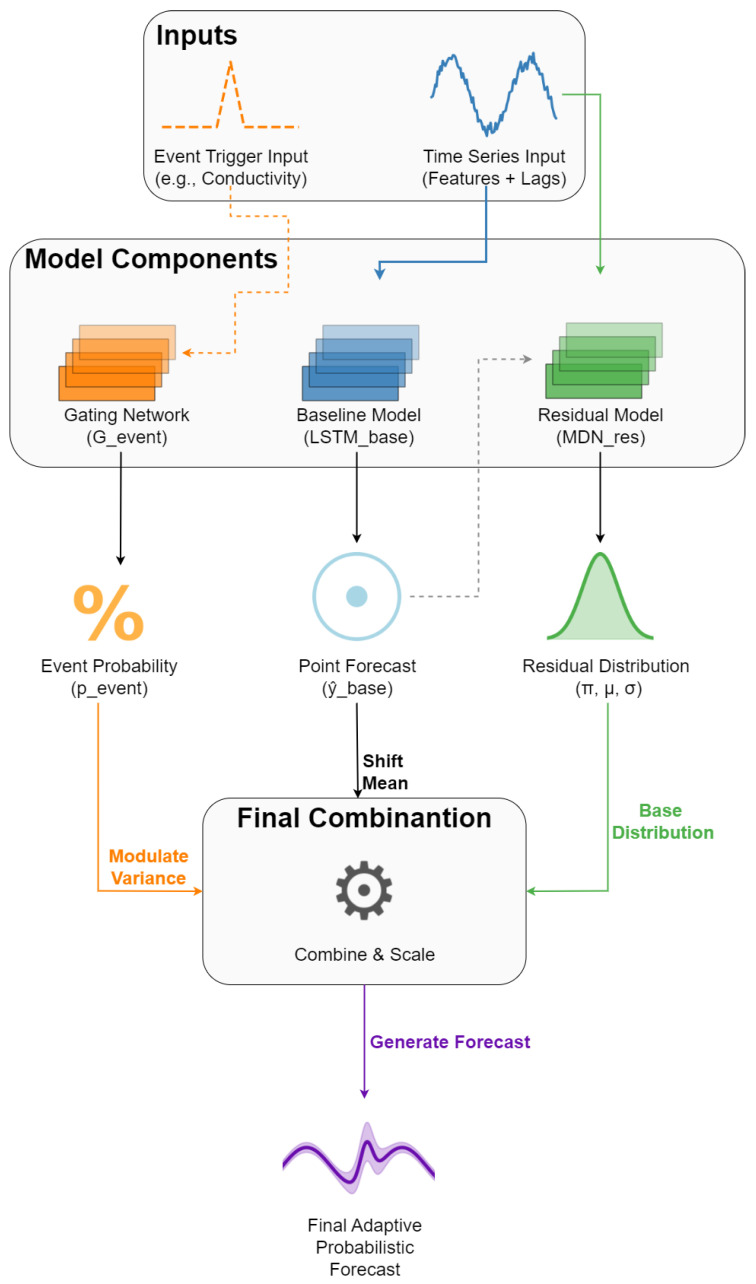
Conceptual architecture of the Gated Hybrid–Mixture Density Network (GH-MDN) framework.

The GH-MDN framework, depicted conceptually in Figure 4, is a hybrid model designed to capture complex, event-driven heteroscedastic patterns in WQ time series.

As shown in Figure 4, the framework is composed of three primary components: the LSTM-Baseline provides an initial point forecast; the Hybrid-MDN-NoGate models the distribution of the baseline’s residuals; and the $G_{event}$ network outputs an event probability, $p_{event}$, which is used to modulate the variance of the residual distribution for the final adaptive forecast.

#### 3.4.1. Baseline LSTM ($LSTM_{base}$)

**Figure 5:**
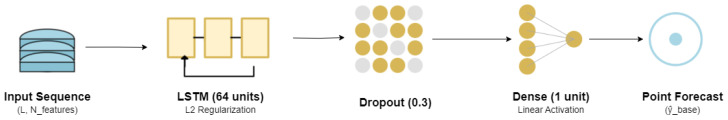
Architecture of the $LSTM_{base}$ component.

The first component is the LSTM-Baseline model, whose architecture is depicted in Figure 5 and consists of:

- An input layer accepting sequences of shape $\left(L,N_{features}\right)$.
- An LSTM layer with 64 units, L2 kernel regularization of 0.001.
- A Dropout layer with a rate of 0.3 for regularization.
- A Dense output layer with a single unit and linear activation to predict the target WQ parameter.

It is trained first to predict the primary trend of the target WQ variable, yielding point forecasts ${\hat{y}}_{base,t+1}$.

#### 3.4.2. Residual Mixture Density Network (${\mathrm{MDN}}_{res}$)

The second component is an MDN that models the distribution of the residuals $r_{t+1}=y_{t+1}-{\hat{y}}_{\mathrm{base},t+1}$. This allows the MDN to focus on the more complex, potentially non-Gaussian and heteroscedastic, part of the signal that the baseline LSTM could not capture. This approach, where a neural network learns the parameters of a conditional distribution, is a foundational technique in the broader field of deep probabilistic modeling, which also includes more powerful, modern architectures like Normalizing Flows [30].

**Figure 6:**
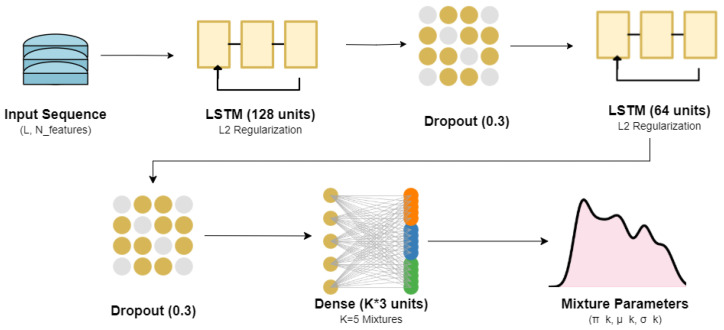
Architecture of the ${\mathrm{MDN}}_{res}$ component.

As shown in Figure 6, the ${\mathrm{MDN}}_{res}$ architecture includes:

- An input layer taking the same input sequences as LSTM-Baseline.
- Two LSTM layers (e.g., 128 units then 64 units, as per Table 2), each with L2 regularization (0.001) and Dropout (0.3).
- A Dense output layer that produces the parameters for *K* mixture components (in our case, K=5 Gaussian distributions). For each component *k*, it outputs a mean $\mu_{k}$, a standard-deviation parameter $\sigma_{k}^{\prime }$, and a mixture weight $\pi_{k}$.
- The custom MDNLayer processes these raw outputs:
  - Mixture weights $\pi_{k}$ are obtained by applying a softmax function to the corresponding raw outputs.
  - Standard deviations $\sigma_{k}$ are obtained by applying a softplus activation to $\sigma_{k}^{\prime }$, adding a small constant ($1\times 10^{-6}$), and then ensuring a minimum value using MDN_MIN_SCALE ($1\times 10^{-3}$ as per Table 2): $\sigma_{k}=\max(\mathrm{softplus}\left(\sigma_{k}^{\prime }\right)+1\;\times \;10^{-6},$ MDN_MIN_SCALE).

The predictive distribution for the residual $r_{t+1}$ is then a mixture of *K* Gaussians:

(1) $$P\left(r_{t+1}|X_{t};\theta_{MDN_{res}}\right)=\sum_{k=1}^{K}\pi_{k}\left(X_{t}\right)N\left(r_{t+1}|\mu_{k}\left(X_{t}\right),\sigma_{k}^{2}\left(X_{t}\right)\right)$$

The ${\mathrm{MDN}}_{res}$ is trained by minimizing the negative log-likelihood (NLL) of the true residuals $r_{t+1}$, given the predicted mixture parameters. The Adam optimizer is used with a learning rate specific to the residual network (e.g., LEARNING_RATE_RESIDUAL in Table 2).

#### 3.4.3. Gating Network (G_event)

**Figure 7:**
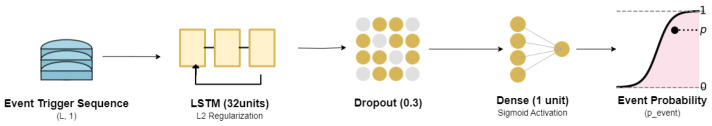
Architecture of the $G_{event}$ component.

The third component, depicted in Figure 7, is a dedicated gating network designed to identify periods of high variability or “events” that might require wider PIs.

- Input: A sequence of the designated event-triggering variable (Conductivity was used for all targets in these experiments; see Table 2). This sequence has the same length *L* as the main model inputs.
- Architecture: A simpler LSTM network (32 units, as per Table 2), with L2 regularization (0.001) and Dropout (0.3), followed by a Dense output layer with a single unit and a sigmoid activation function. This output, $p_{event}\in \left[0,1\right]$, represents the probability of being in an “event” state.
- Training Target: The $G_{event}$ network is trained as a binary classifier. The target labels are derived from the residuals of the LSTM-Baseline model on the training data. A residual $|r_{train}|$ exceeding a certain percentile (90% or 95% based on experimental sweeps, see Table 2) of all absolute training residuals is labeled as an event (1), otherwise, a non-event (0).
- Loss and Optimization: The $G_{event}$ is trained using binary cross-entropy loss and the Adam optimizer with a specific learning rate (e.g., LEARNING_RATE_GATING).

#### 3.4.4. GH-MDN Inference and Dynamic Gating

Once the three components are trained according to the sequential strategy described in Section 3.5, they are integrated into a unified inference pipeline. During forecasting, the outputs are combined dynamically to produce the final probabilistic prediction.

The gating network’s probabilistic output, $p_{event}\in \left[0,1\right]$, is used to directly modulate the variance of the ${\mathrm{MDN}}_{res}$ components. This is achieved through a soft-gating mechanism, where a scale multiplier, $m_{scale}$, is defined as:

(2) $$m_{scale}=1.0+p_{event}\cdot \left(\mathrm{GATE}\_\mathrm{VARIANCE}\_\mathrm{MULTIPLIER}-1.0\right)$$

where GATE_VARIANCE_MULTIPLIER is the hyperparameter determining the maximum amplification of the variance during an event (set to 5.0 in our experiments). Unlike a hard-gating switch based on a threshold, this soft-gating approach allows the variance to scale smoothly with the gating network’s confidence.

The final predictive distribution for $y_{t+1}$ is constructed by shifting the residual distribution by the baseline’s point forecast and expanding its width based on the gate:

(3) $$P\left(y_{t+1}|\cdot \right)=\sum_{k=1}^{K}\pi_{k}N\left(y_{t+1}|{\hat{y}}_{base,t+1}+\mu_{k},{\left(m_{scale}\cdot \sigma_{k}\right)}^{2}\right)$$

Here, $\pi_{k},\mu_{k},\sigma_{k}$ are the parameters predicted by the residual MDN, and ${\hat{y}}_{base,t+1}$ is the deterministic point forecast from the baseline component. This formulation allows the model to retain high precision during stable periods while seamlessly expanding its uncertainty envelope during detected events.

#### 3.4.5. Hybrid MDN Without Gating (Hybrid-MDN-NoGate)—Ablation Study

To assess the specific contribution of the gating mechanism, we include an ablation model called Hybrid-MDN-NoGate. This model is identical to the GH-MDN but with the scale multiplier $m_{scale}$ fixed to 1.0. Its predictive distribution is:

(4) $$P\left(y_{t+1}|\cdot \right)=\sum_{k=1}^{K}\pi_{k}N\left(y_{t+1}|{\hat{y}}_{base,t+1}+\mu_{k},\sigma_{k}^{2}\right)$$

### 3.5. Training Procedure

The optimization of the GH-MDN framework is conducted using a sequential curriculum learning strategy. Unlike end-to-end approaches where gradients flow through all components simultaneously, this sequential protocol isolates the learning objectives for the deterministic baseline, the residual distribution, and the event gating mechanism. This decomposition ensures that the gating network learns to react specifically to the failure modes of a fully converged baseline, rather than co-adapting to a moving target.

Consequently, unless explicitly stated otherwise (as in the comparative analysis in Section 5.7), the sequential training procedure detailed in Algorithm 1 constitutes the standard protocol employed for all experimental results and performance benchmarks reported in this study.

All models use EarlyStopping (monitoring validation loss, patience based on epoch settings) and ReduceLROnPlateau callbacks during training. The number of epochs for each component and learning rates are key hyperparameters, detailed in Table 2.
**Algorithm 1** Sequential Training Curriculum for GH-MDN (Conceptual)**Require:** Training data $D_{train}={\left\{\left(X_{t},y_{t}\right)\right\}}_{t=1}^{N}$, Gating Input $X_{g}$**Ensure:** Optimized parameters $\theta_{base}$, $\theta_{res}$, $\theta_{gate}$   **Phase 1:** **Deterministic Trend Learning** 1:Initialize ${\mathrm{LSTM}}_{base}$ parameters $\theta_{base}$ 2:$\theta_{base}^{*}\leftarrow {\mathrm{argmin}}_{\theta }L_{\mathrm{MSE}}\left(y,f_{base}\left(X;\theta \right)\right)$ 3:Generate point forecasts: $\hat{y}\leftarrow f_{base}\left(X;\theta_{base}^{*}\right)$ 4:Compute residuals: $r\leftarrow y-\hat{y}$   **Phase 2:** **Residual Distribution Learning** 5:Freeze $\theta_{base}^{*}$ 6:Initialize ${\mathrm{MDN}}_{res}$ parameters $\theta_{res}$ 7:$\theta_{res}^{*}\leftarrow {\mathrm{argmin}}_{\theta }-\sum \log P\left(r|X;\theta \right)$     ▹Minimize Negative Log-Likelihood   **Phase 3:** **Event Gating Learning** 8:**if** Gating Enabled **then** 9:     Define event threshold τ←Percentile(|r|,95)  10:     Generate binary targets: $y_{gate}\leftarrow I\left(|r|>\tau \right)$  11:     Initialize $G_{event}$ parameters $\theta_{gate}$  12:     $\theta_{gate}^{*}\leftarrow {\mathrm{argmin}}_{\theta }L_{\mathrm{BCE}}\left(y_{gate},f_{gate}\left(X_{g};\theta \right)\right)$  13:**return** Combined Inference Model $M(\theta_{base}^{*},\theta_{res}^{*},\theta_{gate}^{*})$

## 4. Experimental Design

### 4.1. Baseline and Benchmark Architectures

#### 4.1.1. Standard Baselines

Two baseline models are used for comparison:

##### LSTM-Baseline 

This is a standard LSTM network designed for point forecasting as described in Section 3.4.1. The model is compiled using the Adam optimizer [31] with a learning rate (e.g., 0.001, see Table 2 for swept values) and Mean Squared Error (MSE) loss.

##### LSTM-ConstantVariance 

This model provides a simple probabilistic baseline. It uses the LSTM-Baseline model to generate point forecasts ${\hat{y}}_{t+1}$. The uncertainty is modeled by assuming a Gaussian distribution with a constant standard deviation $\sigma_{res}$. This $\sigma_{res}$ is estimated empirically as the standard deviation of the residuals ($y_{\mathrm{val}}-{\hat{y}}_{\mathrm{val}}$) on the validation set:

(5) $$P\left(y_{t+1}|\cdot \right)=N\left({\hat{y}}_{base,t+1},\sigma_{res}^{2}\right)$$

While simple, this approach does not account for heteroscedasticity.

#### 4.1.2. Advanced Benchmark: Multi-Task M2P-MDN Hybrid

To further validate the performance of the proposed GH-MDN framework, we conducted a comprehensive benchmark against several advanced probabilistic forecasting models, following the best practice of evaluating new methods against a suite of strong baselines on challenging, real-world data [32]. This evaluation extends beyond the initial baselines to include: (1) a Deep Ensemble of LSTMs, a robust and widely used method for uncertainty quantification; (2) a Quantile Regression LSTM (LSTM-QR), which directly forecasts prediction interval bounds; and (3) a novel M2P-MDN Hybridarchitecture.

**Figure 8:**
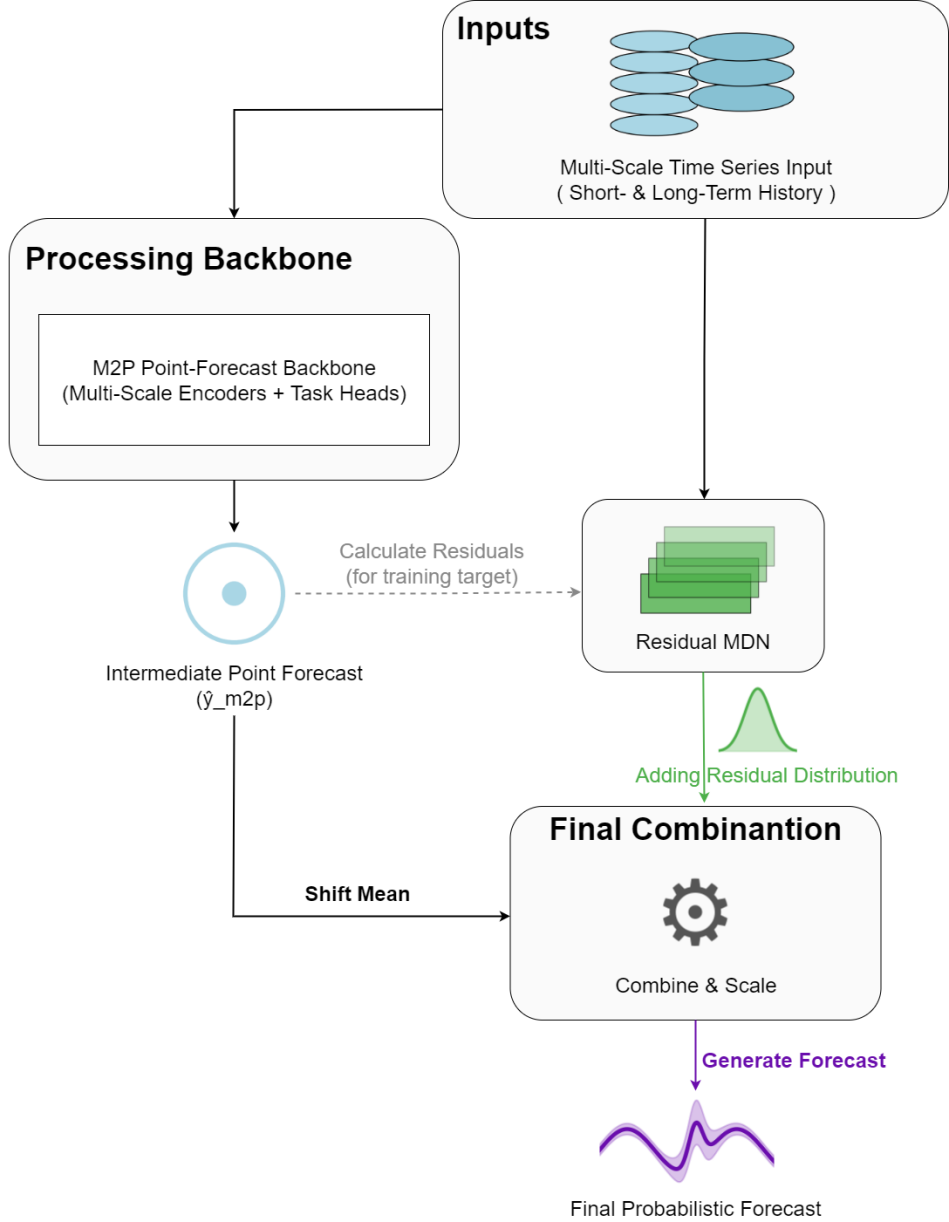
Conceptual architecture of the M2P-MDN Hybrid benchmark model.

This hybrid benchmark model, depicted in Figure 8, is designed to test an alternative architectural philosophy based on residual modeling. It is composed of two main stages. The first stage is the M2P Point-Forecast Backbone, an interpretable multi-task model that generates a deterministic forecast from the historical inputs. This backbone explicitly disentangles processes occurring on different timescales by using parallel encoders for short-term and long-term history, allowing it to separate event dynamics from slower sensor drift. The second stage is a Residual MDN, which takes the original inputs and the errors from the M2P’s intermediate forecast to model the remaining uncertainty, ultimately producing a full probabilistic distribution.

### 4.2. Prediction-Interval Generation and Calibration

For models that output a predictive distribution (LSTM-ConstantVariance, Hybrid-MDN-NoGate, GH-MDN), PIs are generated by sampling from this distribution. For a (1−α)×100% PI, the α/2 and 1−α/2 quantiles of $N_{\mathrm{samples}}$ (e.g., 10,000) drawn from the predictive distribution form the lower and upper bounds. The median of these samples is used as the point forecast for MAE/RMSE calculation for these probabilistic models.

Raw PIs from deep-learning models are often uncalibrated, a well-documented issue with modern, high-capacity networks [33]. We investigate two post hoc calibration strategies applied to the validation set predictions to adjust PIs on the test set:

1. Confidence-Level Calibration: The nominal confidence level is adjusted to achieve a target PICP (0.95) on the validation data. This adjusted level is then used for generating PIs on the test set.
2. Quantile Scaling-Factor Calibration: For a fixed nominal confidence level, a scaling factor $q_{scale}$ is found that adjusts the width of the raw PIs such that the target PICP is met on the validation data. This $q_{scale}$ is then applied to test set PIs.

The primary results reported in this paper utilize PIs derived from the confidence-level calibration strategy.

### 4.3. Evaluation Metrics

Model performance is assessed using a suite of deterministic and probabilistic metrics:

- Mean Absolute Error (MAE): Measures the average absolute difference between true values and point forecasts.
- Root Mean Squared Error (RMSE): Measures the square root of the average squared difference.
- Continuous Ranked Probability Score (CRPS): A proper scoring rule that generalizes the Mean Absolute Error across the entire predictive distribution, providing a single score that assesses both calibration and sharpness. As with MAE, a lower CRPS value indicates a better forecast.
- Prediction Interval Coverage Probability (PICP): The proportion of true observations that fall within the PIs. For well-calibrated PIs, PICP should be close to the nominal confidence level (e.g., 0.95).
- Mean Prediction Interval Width (MPIW): The average width of the PIs. Sharper (narrower) PIs are preferred, given that PICP is maintained.

Reliability Diagrams are also used to visually assess PI calibration by plotting observed PICP against a range of nominal confidence levels [34]. Ideally, points should lie on the diagonal.

### 4.4. Implementation Details

All models were implemented using Python 3.10 with TensorFlow (v2.x) and TensorFlow Probability (TFP v0.x). Key hyperparameters used for the main experiments, including swept values, are summarized in Table 2. These were chosen based on preliminary experiments and common practices. The hyperparameter ranges for sweeping, particularly for learning rates (0.001 and 0.0005) and the gate event threshold percentile (90% and 95%), were selected to explore standard values for the Adam optimizer and to test reasonable definitions of an ‘event’ as a rare but significant deviation from the norm. The primary experiments involved sweeps over these learning rates and percentiles.

A 5-fold TimeSeriesSplit cross-validation strategy was employed (as per CMD ‘–run-cv 5’). For each fold, the data is split chronologically into training, validation, and test sets. The validation set is used for early stopping, learning-rate reduction, and PI calibration, while the test set is used for final performance evaluation. This ensures that the models are evaluated on unseen future data, respecting the temporal order of observations.

## 5. Results and Discussion

### 5.1. Overall Performance vs. Baselines

The aggregated performance metrics across all 5 cross-validation folds are summarized in Table 3. These results highlight the foundational improvements offered by the MDN-based architectures over simpler baselines.

Two key observations emerge from this initial comparison. Firstly, for point forecast accuracy (MAE and RMSE), the MDN-based models (Hybrid-MDN-NoGate and GH-MDN), using the median of their predictive distributions, generally achieve values comparable to or slightly better than the dedicated point-forecasting LSTM-Baseline.

Secondly, and more importantly for probabilistic forecasting, both MDN-based models consistently and significantly outperform the LSTM-ConstantVariance model across all target variables in terms of CRPS. For example, for Turbidity, the MDN models achieve a CRPS of around 0.005, while LSTM-ConstantVariance has a much higher CRPS of 0.0115. This indicates that the MDNs’ ability to model a flexible distribution provides a much better representation of the true underlying uncertainty. The LSTM-ConstantVariance, which assumes a constant variance Gaussian, is too simplistic for these complex WQ time series and serves as a poor probabilistic baseline.

The statistical significance of the results was confirmed using a paired Wilcoxon signed-rank test (N=400). As detailed in Table 4, the analysis yielded a *p*-value p≪0.001, allowing us to reject the null hypothesis that the models perform equally. The 95% bootstrap confidence interval for the improvement in CRPS [0.0032,0.0040] lies entirely above zero, demonstrating that the GH-MDN architecture provides a robust and strictly positive improvement in probabilistic calibration and sharpness across varying stations and targets.

### 5.2. Effectiveness of the Gating Mechanism: Gated vs. Non-Gated MDN

Having established the superiority of the MDN approach, we now “zoom in” on the core research question: the specific contribution of the proposed gating mechanism. Table 5 presents the head-to-head results of the final, tuned GH-MDN and Hybrid-MDN-NoGate models.

The results in Table 5 illustrate the same limitation identified in the introduction: CRPS alone does not adequately reflect performance in event-driven time series. The Hybrid-MDN-NoGate achieves a slightly lower CRPS, which is consistent with the known tendency of aggregate metrics to reward narrow, over-confident predictive intervals during long periods of stability. By keeping its uncertainty artificially tight, the non-gated model accumulates a low error score in calm conditions but fails to cover the events that matter most. This trade-off is further demonstrated in the visual forecast plots.

In contrast, the GH-MDN adapts its predictive spread during extremes. Although this behavior slightly increases the aggregate CRPS, it yields forecasts that remain reliable and well calibrated—properties essential for operational warning systems. From this perspective, the GH-MDN provides the more trustworthy architecture, despite the small difference in raw CRPS.

**Figure 9:**
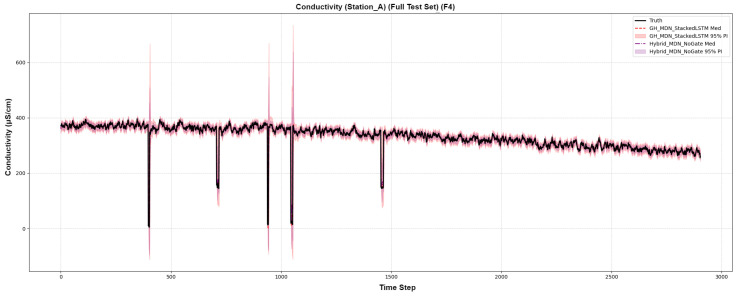
Forecast comparison on a volatile conductivity series.

The visual evidence from forecast plots provides definitive proof of the gating mechanism’s value. For a volatile series like Conductivity (Figure 9), the Hybrid-MDN-NoGate (purple PI) fails to expand to cover extreme spikes. In stark contrast, the GH-MDN (red PI) dynamically and dramatically widens at the exact moments the spikes occur, successfully capturing the event. This visually proves that the gating mechanism is working precisely as designed.

**Figure 10:**
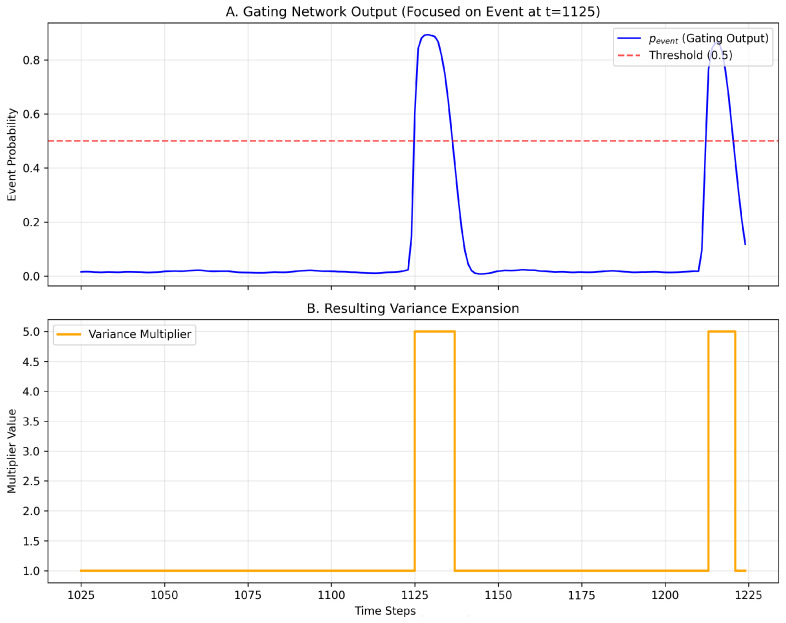
Temporal correlation between the Gating Network’s event probability output ($p_{event}$) and the variance expansion multiplier.

To quantitatively verify the gating mechanism’s behavior, we analyzed the temporal correlation between the event probability ($p_{event}$) and the variance multiplier. As illustrated in Figure 10, the gating network maintains a low probability ($p_{event}\approx 0.02$) during stable conditions, resulting in a standard variance multiplier of 1.0. However, during specific intervals (e.g., t≈1125), $p_{event}$ sharply rises above the 0.5 threshold, triggering an immediate step-change in the variance multiplier to 5.0. Crucially, this activation coincides with a rapid negative excursion in the Conductivity signal. This pattern aligns with the hydrological ‘dilution effect’, where the influx of low-ionic-strength storm runoff dilutes the mineral-rich baseflow, causing a sharp drop in conductivity. Consistent with findings by [35], who observed that extreme flood events drive a distinct dilution pattern in rock weathering products during the rising limb, the gating network has effectively learned to utilize this drop in ionic concentration as a physical proxy for the onset of turbulent, high-energy mixing. Consequently, the model recognizes that the system has shifted from a stable, groundwater-dominated regime to a volatile, runoff-dominated state, and appropriately expands the uncertainty envelope. This confirms that the model successfully identifies distinct temporal features associated with higher uncertainty and dynamically adjusts the predictive distribution width in response.

**Figure 11:**
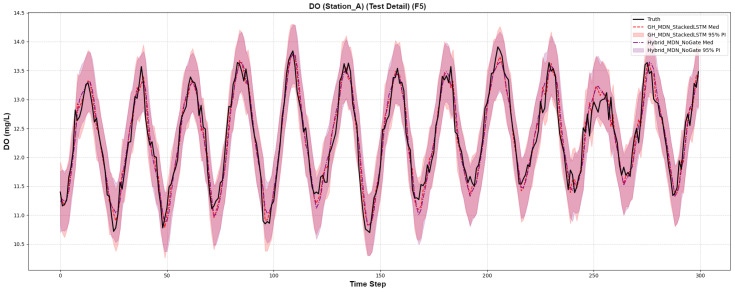
Forecast comparison on a smooth dissolved oxygen series.

For a smoother series like Dissolved Oxygen (Figure 11), where there are no major “events”, the PIs for both models are more similar. The GH-MDN’s interval is slightly wider, aligning with the MPIW numbers in Table 5 and reflecting its more conservative nature.

### 5.3. Analysis of Prediction Interval Calibration

**Figure 12:**
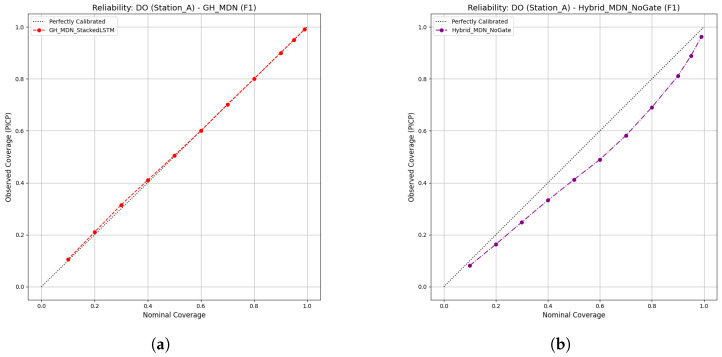
Reliability Diagrams for Gated and Non-Gated Models. (**a**) GH-MDN (Near-perfect calibration), (**b**) Hybrid-MDN-NoGate (Over-confident).

Reliability diagrams provide a powerful argument for the superiority of the gated model’s uncertainty quantification. While the LSTM-ConstantVariance model typically shows poor calibration, a more telling comparison is between the two advanced MDN models. As shown in Figure 12, the GH-MDN is exceptionally well calibrated. Its curve lies almost perfectly on the diagonal, indicating that its predicted probabilities are highly reliable.

Conversely, the Hybrid-MDN-NoGate model’s curve is consistently below the diagonal. This indicates the model is over-confident: its prediction intervals are systematically too narrow, and the true value falls outside them more often than the model’s own probabilities would suggest. This analysis reveals a critical insight: the GH-MDN is not only adaptive but also produces more honest and trustworthy probabilistic forecasts.

The near-perfect calibrated PICP values for the non-gated model in Table 5 should be interpreted with caution. They are an artifact of a strong post hoc calibration process that forces the empirical coverage to match the target. The reliability diagram (Figure 12b), which assesses intrinsic calibration without this adjustment, reveals the model’s underlying flaw of over-confidence. The superior intrinsic calibration of the GH-MDN means its excellent performance is more inherent and less dependent on such post-processing.

### 5.4. Real-World Case Study Validation

We trained our two primary models, Hybrid-MDN-NoGate and GH-MDN, on the full VUB dataset (detailed in Section 3.2.2) and evaluated their performance on a held-out test set.

**Figure 13:**
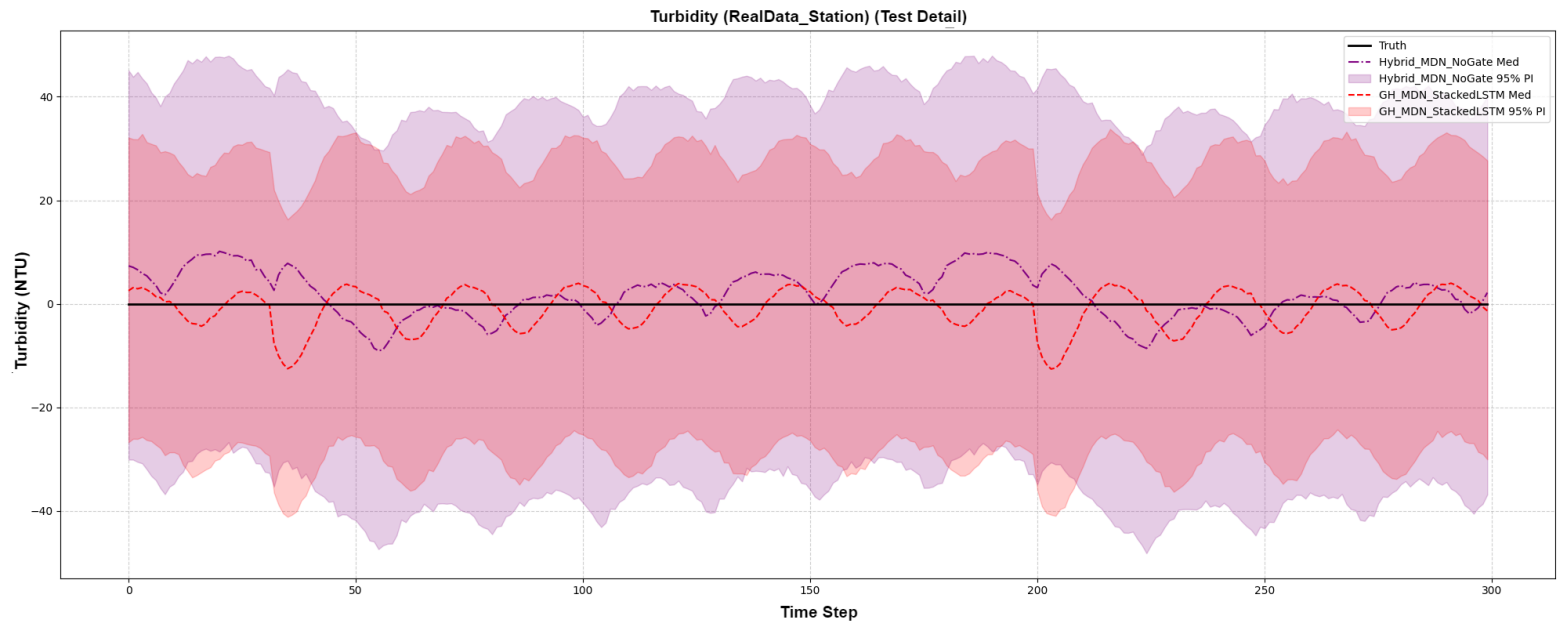
Probabilistic forecast for Turbidity on the real-world case study dataset, comparing the gated (GH-MDN) and non-gated (Hybrid-MDN-NoGate) models.

The results, shown in Figure 13, provide compelling evidence of the gating mechanism’s ability to adapt to changing conditions. The test period for this dataset proved to be relatively calm, without the extreme spikes seen in our synthetic event data. In this scenario, the non-gated model, having learned about volatility from the training set, produced a single, overly conservative prediction interval that was unnecessarily wide throughout the entire test period.

In stark contrast, the GH-MDN model’s gating network correctly identified the calm conditions and actively suppressed the predictive variance. This resulted in a forecast that was not only significantly more accurate on all metrics (e.g., CRPS of 0.0027 vs. 0.0036) but also produced a sharper, more confident prediction interval (MPIW of 0.0403 vs. 0.0531). This case study demonstrates a crucial aspect of the proposed architecture: the gating mechanism is not merely an “on-switch” for high-variance events but an adaptive “dimmer switch” that improves precision and reduces unnecessary conservatism during stable periods. This confirms the model’s ability to generate more appropriate and trustworthy uncertainty estimates in a real-world setting.

### 5.5. Generalizability and Comparative Benchmarking

#### 5.5.1. Comparative Evaluation and Results

Having established the internal mechanics and calibration benefits of the gating mechanism on both synthetic and the initial real-world case study, we now challenge the GH-MDN framework against a broader set of rigorous benchmarks on a completely independent real-world dataset. This step is crucial to ensure that the model’s performance is not an artifact of the datasets encountered thus far but represents a generalized ability to handle event-driven volatility. To this end, we utilized the Kenyan River Dataset (defined in Section 3.2.3) against the M2P-MDN Hybrid and other probabilistic models (defined in Section 4.1.2).

**Figure 14:**
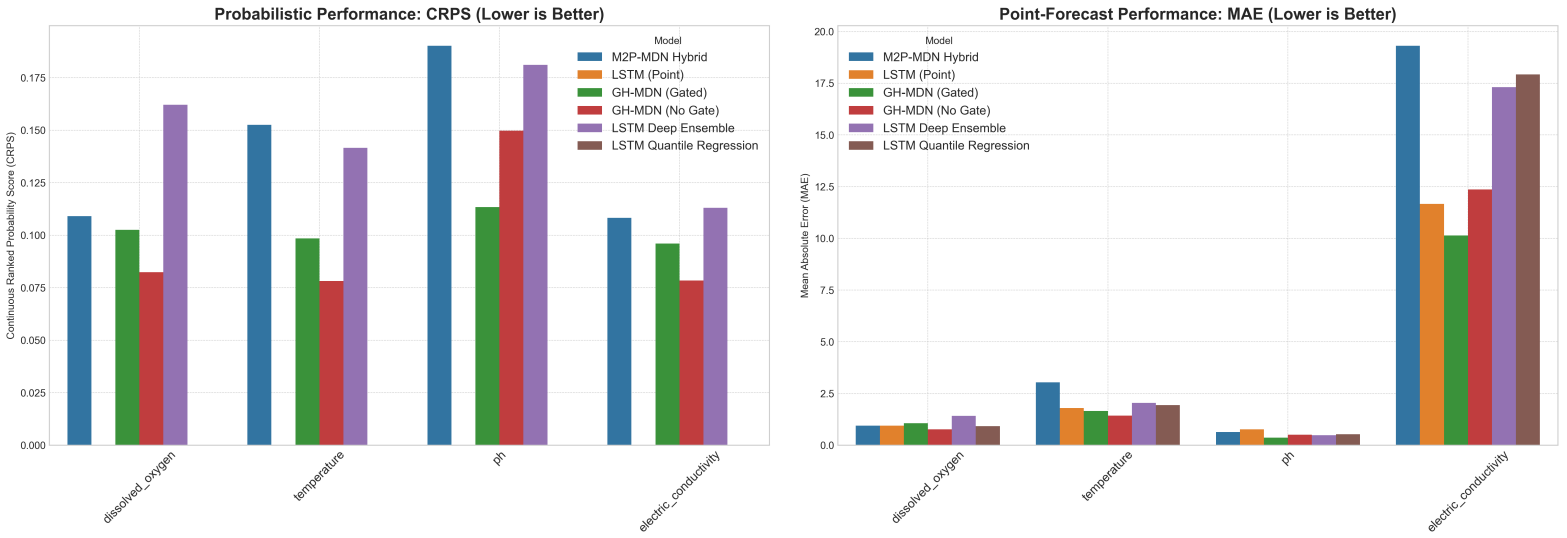
Benchmark performance comparison across all models and targets.

The comparative results from a 5-fold time-series cross-validation are presented in Figure 14. The left panel shows the Continuous Ranked Probability Score (CRPS), where lower values indicate better probabilistic forecasts. The right panel shows the Mean Absolute Error (MAE), where lower values indicate better point-forecast accuracy. The results clearly show the superior probabilistic performance of the MDN-based models.

#### 5.5.2. MDN Architectures Demonstrate Superior Probabilistic Acuity

The left panel of Figure 14 clearly illustrates the superior performance of all three MDN-based architectures (GH-MDN, Hybrid-MDN-NoGate, and M2P-MDN Hybrid) on the CRPS metric. For every target variable, these specialized models significantly outperform the LSTM Deep Ensemble, which serves as a strong but more generalized probabilistic baseline. For instance, in forecasting dissolved oxygen, the MDN models achieve a CRPS approximately 30–50% lower than the Deep Ensemble, highlighting their enhanced ability to learn the true underlying predictive distribution.

#### 5.5.3. The Critical Role of the Gating Mechanism

A direct comparison between the green bars (GH-MDN (Gated)) and red bars (Hybrid-MDN -NoGate) reveals the crucial trade-off between aggregate performance and reliability. For volatile parameters like electric conductivity, the non-gated model achieves the lowest CRPS, indicative of an over-confident model that excels in stable conditions but, as demonstrated in our earlier analysis, fails to provide adequate risk coverage during events. The gated model, while showing a slightly higher CRPS, provides more honest and well calibrated uncertainty estimates. This confirms that the explicit, learnable gating mechanism is essential for producing trustworthy and reliable forecasts in operational settings where capturing extreme events is critical.

#### 5.5.4. Architectural Trade-Offs and the M2P-MDN Hybrid

The novel M2P-MDN Hybrid (blue bars) establishes itself as a top-tier probabilistic forecaster. Its CRPS scores are highly competitive with the other MDN models, and its unique architecture—which explicitly disentangles long-term drift from short-term events—offers a promising path toward more interpretable deep learning models for water quality.

To provide a broader context for our model’s point-forecast capabilities, we compared the MAE of the GH-MDN against a state-of-the-art, multi-task, process-disentangled (M2P) architecture designed for interpretability. The results, shown in Table 6, highlight an interesting trade-offs in forecasting accuracy. While the GH-MDN provides superior probabilistic forecasts, the M2P’s design shows strength on specific event-driven variables.

While our GH-MDN achieves a superior MAE on pH and provides the well-calibrated probabilistic forecasts that are the primary goal of this work, the M2P architecture’s focus on disentangling events allows it to achieve a lower point-forecast error on the highly volatile DO and EC variables. This suggests that for applications where pure deterministic accuracy on event-driven signals is paramount, a multi-task approach is a powerful alternative, confirming that the optimal architecture is highly dependent on the specific forecasting objective.

#### 5.5.5. Summary of Findings

This extended benchmark confirms that for the complex task of event-driven water quality forecasting, specialized architectures are superior to general-purpose probabilistic methods. While the non-gated MDN model can appear best on aggregate metrics, its inherent overconfidence makes it less suitable for critical applications. The GH-MDN proves to be the most robust and reliable framework, providing the best balance of probabilistic accuracy and adaptive, trustworthy uncertainty quantification, thereby validating it as the superior model for real-world environmental management.

### 5.6. Analysis of Computational Cost

To assess the practical viability of the proposed models, we analyzed their computational cost in terms of both training and inference time. Table 7 summarizes these costs alongside the CRPS performance for the Turbidity target, averaged over 5 cross-validation folds for a learning rate of 0.001. Training and inference were performed on a CPU, and the times represent the duration for a single fold.

As expected, the more complex MDN-based models, such as GH-MDN, require a significantly longer training time (an average of 539.62 s per fold) compared to the simpler LSTM-Baseline (104.45 s). This reflects the increased number of parameters and the complexity of the unified negative log-likelihood loss calculation.

Critically, however, the inference time—the duration required to generate forecasts for the entire test set of a fold—remains highly practical for all models. For instance, the inference time for the GH-MDN model was approximately 2.88 s, demonstrating its suitability for real-world, operational scenarios where timely forecasts are essential. This analysis shows that the substantial gains in predictive performance and reliability offered by our proposed model come at a justifiable one-time training cost, while maintaining a fast and efficient inference speed for generating predictions.

### 5.7. Comparison of Training Strategies: Sequential vs. End-to-End

The primary training procedure described in this work is sequential: a baseline LSTM model is trained first, its weights are frozen, and a residual MDN is subsequently trained on the resulting errors. While this modular approach is straightforward and effective, it is theoretically possible that it leads to a sub-optimal solution, as the components are trained independently and cannot co-adapt.

To investigate this theoretical concern and explore a potentially more robust training regimen, we also implemented and evaluated a fully end-to-end framework for the GH-MDN model. In this alternative strategy, the baseline LSTM, the residual MDN, and the gating network are constructed as a single, unified model. The entire architecture is trained simultaneously by optimizing a single negative log-likelihood (NLL) loss function. This allows gradients from the final probabilistic output to flow back through all components, enabling them to learn their respective parameters cohesively.

A direct comparison was conducted using the same 5-fold time-series cross-validation setup on the volatile Turbidity target. The results, summarized in Table 8, show that the end-to-end training method achieves a mean CRPS on the Turbidity target that is highly competitive with the sequential approach.

This result is significant: it demonstrates that the more theoretically sound and unified end-to-end training framework can be adopted without a loss in predictive performance. This validates the robustness of the proposed architecture and offers a more elegant, single-stage training procedure for practical implementation.

### 5.8. Hyperparameter Sensitivity

**Figure 15:**
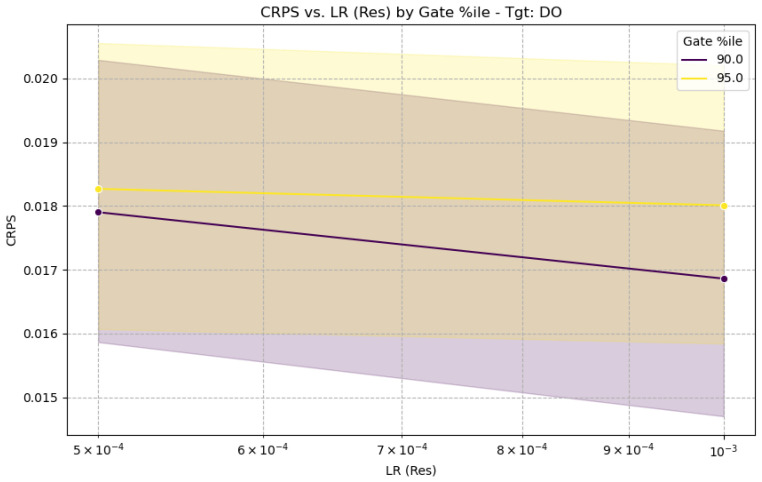
CRPS sensitivity to learning rate and gating percentile.

**Figure 16:**
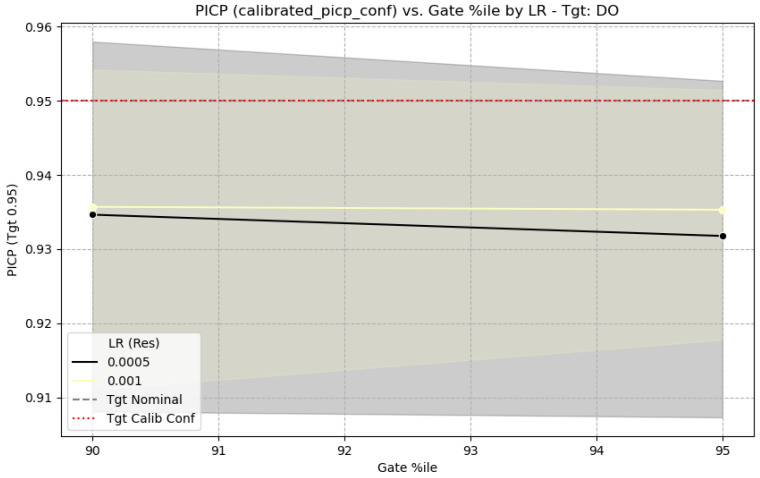
PICP sensitivity to learning rate and gating percentile.

The performance of the GH-MDN can be sensitive to certain hyperparameters, particularly the learning rate of the residual network (LR (Res)) and the GATE_EVENT_THRESHOLD_ PERCENTILE used to define events for training the gating network. Figure 15 shows the sensitivity of CRPS for DO to these parameters, and Figure 16 shows the sensitivity of calibrated PICP, based on the full experiments.

From Figure 15, for DO, a learning rate of 0.001 for the residual network generally provides good CRPS values, with performance degrading slightly at 0.0005. The choice of gating percentile (90% vs. 95%) shows some interaction, with 95% sometimes yielding slightly better CRPS with LR 0.001 for DO. Figure 16 indicates that the calibrated PICP remains relatively stable around the target 0.95 across different gating percentiles when a suitable learning rate is used, suggesting the calibration process is robust to these choices to some extent. The selection of $LR_{res}=0.001$ and GATE_EVENT_THRESHOLD_PERCENTILE values around 90–95% for the main reported results (Table 5) is justified by these sensitivity analyses as a reasonable balance, although target-specific tuning could yield further improvements.

### 5.9. Discussion of MDN Component Behavior

Inspection of the MDN parameters during training and inference (e.g., “Validation Set MDN Parameter Stats”) revealed dynamic behavior. The logits ($\pi_{k}$), locations ($\mu_{k}$), and scales ($\sigma_{k}$) of the Gaussian mixture components varied over time and across different input sequences, confirming that the ${\mathrm{MDN}}_{res}$ was learning complex conditional distributions. The MDN_MIN_SCALE parameter (set to $1\times 10^{-3}$ in these experiments, see Table 2) played an important role in preventing numerical instability by ensuring that predicted standard deviations did not collapse to zero, which can be an issue in MDN training, especially with highly peaked residual distributions. The softplus activation for scales, combined with this minimum, ensured positive and non-trivial variance predictions. For example, in the experiments.csv related ‘log.txt’ snippet for Turbidity Fold 1, (LR 0.001, Perc 90, Hybrid-MDN-NoGate), ‘Scales_final’ on the validation set shows a ‘Min: $2.8807\times 10^{-3}$’, which is above the configured MDN_MIN_SCALE of $1\times 10^{-3}$, indicating the MDN learned appropriate scales.

## 6. Conclusions and Future Work

In this work, we addressed the critical challenge of modeling event-driven heteroscedasticity in complex environmental systems. We proposed the GH-MDN, a novel framework whose architecture is explicitly designed to mirror the underlying physical processes: a deterministic baseline, a stochastic residual, and an event-triggered variance component. Through a comprehensive evaluation on a challenging synthetic dataset and validation on a real-world case study, we have demonstrated its practical superiority over simpler baselines and its non-gated counterpart.

Our comprehensive evaluation, informed by a rigorous, review-informed tuning process, yielded several key insights. We confirmed that MDN-based models are essential for flexibly capturing the complex uncertainty in WQ data, substantially outperforming simpler constant-variance models. Furthermore, while a non-gated model can achieve competitive aggregate scores, this comes at the cost of being poorly calibrated and failing to provide reliable risk coverage during critical events. The proposed GH-MDN addresses this directly. Our analysis, including the real-world case study and a quantitative dissection of interval widths, proves that the gating mechanism is not a blunt instrument but an adaptive ‘dimmer switch’ that intelligently modulates variance. This confirms that the model is not only more reliable but also more trustworthy.

The main contributions of this work are therefore twofold: the novel GH-MDN architecture itself, and the detailed analysis demonstrating that a carefully tuned, science-informed gating mechanism is the key to balancing probabilistic accuracy with the crucial, practical demands of reliability and adaptive risk management.

For water-resource managers, the distinction between the proposed GH-MDN and a technically proficient but over-confident model like Hybrid-MDN-NoGate has critical operational implications. An over-confident model might forecast an event with a deceptively narrow prediction interval, leading a facility operator to underestimate the risk and delay preventative action. In contrast, by producing a wide, well-calibrated PI during the onset of a storm, the GH-MDN provides an honest and trustworthy signal of high risk and uncertainty. This enables managers to act decisively—for example, by preemptively adjusting treatment processes, diverting initial storm flows, or issuing more timely public health advisories—with the confidence that their actions are justified by a reliable assessment of the potential outcomes.

While the core findings of this study were validated on both synthetic and real-world datasets, further validation on diverse catchments with different hydrological regimes is necessary to fully establish its generalizability. Our sensitivity analysis confirmed that hyperparameters for the gating mechanism—particularly the GATE_VARIANCE_MULTIPLIER and the choice of EVENT_TRIGGER_VARIABLE—are critical and benefit from target-specific tuning. Future work should therefore focus on developing a more systematic framework for selecting these parameters based on the unique physical and chemical drivers of each target variable. Other promising avenues include investigating end-to-end training and enhancing the interpretability of the MDN and gating components for WQ managers. Also, the integration of distribution-free uncertainty quantification methods, such as Conformal Prediction, which can provide rigorous, guaranteed coverage rates for prediction intervals without distributional assumptions [36].

Our benchmark against a multi-task architecture further underscored this, showing that while our specialized probabilistic model provides superior uncertainty quantification, alternative frameworks can excel in point-forecast accuracy for specific event-driven variables.

In summary, the GH-MDN framework presents a promising direction for developing more reliable and adaptive probabilistic WQ forecasting systems. By explicitly modeling event-driven heteroscedasticity, it takes a crucial step towards creating AI systems that are not only accurate, but also transparent and trustworthy, ultimately leading to more robust environmental management and better protection of public health.

## Figures and Tables

**Table 1:** Key Lead–Lag Relationships in the Benchmark Dataset.

Target Variable	Potential Trigger Variable	Peak Correlation	Lag (Hours)
electric_conductivity	temperature	0.61	−36
ph	temperature	0.59	−1
dissolved_oxygen	electric_conductivity	−0.42	1
dissolved_oxygen	ph	0.61	0

**Table 2:** Key configuration and hyperparameter values for the main experiments.

Parameter	Value
Data File	synthetic_river_wq_hourly_deep_learning.csv
Target Variables	Turbidity, pH, Temp, Conductivity, DO
Event Trigger Variable	Conductivity (used for all targets)
Sequence Length (*L*)	72 h
Batch Size	64
Epochs (Baseline/Residual/Gating)	40/100/30
Learning Rates (Baseline/Residual/Gating)	Swept: 0.001/0.001/0.001 or 0.0005/0.0005/0.0005
MDN No. Mixtures (*K*)	5
LSTM Units (Baseline)	64
LSTM Units (Residual L1/L2)	128/64
LSTM Units (Gating)	32
Dropout Rate	0.3
L2 Regularization	0.001
Confidence Level (Nominal)	0.95
PI Calibration Target PICP	0.95
Gate Event Threshold Percentile	Swept: 90% or 95%
Gate Variance Multiplier ($M_{var}$)	5.0
MDN Min Scale	$1\times 10^{-3}$
Include Exogenous Features	True (Rainfall, Flow if available)

**Table 3:** Aggregated performance metrics for all models across 5 cross-validation folds. Best mean value per target is bolded (lower is better).

Target Variable	Model	MAE	RMSE	CRPS	Calib. PICP	Calib. MPIW (Scaled)
Turbidity	LSTM-Baseline	2.3712 ± 0.1827	13.4803 ± 2.1240	–	–	–
	LSTM-ConstantVariance	2.3676 ± 0.1586	13.5281 ± 2.0904	0.0115 ± 0.0031	0.9640 ± 0.0089	0.0177 ± 0.0063
	Hybrid-MDN-NoGate	**1.9475 ± 0.2295**	13.5544 ± 2.2121	**0.0051 ± 0.0021**	0.9719 ± 0.0095	0.0160 ± 0.0050
	GH-MDN	1.9528 ± 0.2242	**13.5327 ± 2.1611**	0.0053 ± 0.0021	0.9806 ± 0.0081	0.0288 ± 0.0088
pH	LSTM-Baseline	0.0150 ± 0.0029	0.0185 ± 0.0033	–	–	–
	LSTM-ConstantVariance	0.0155 ± 0.0031	0.0192 ± 0.0037	0.0076 ± 0.0017	0.9144 ± 0.0615	0.0463 ± 0.0058
	Hybrid-MDN-NoGate	0.0132 ± 0.0027	0.0165 ± 0.0034	**0.0060 ± 0.0014**	0.9577 ± 0.0152	0.0431 ± 0.0048
	GH-MDN	**0.0129 ± 0.0024**	**0.0162 ± 0.0030**	0.0067 ± 0.0019	0.9883 ± 0.0075	0.0591 ± 0.0083
Temp	LSTM-Baseline	0.3187 ± 0.2238	0.3867 ± 0.2607	–	–	–
	LSTM-ConstantVariance	0.2881 ± 0.1911	0.3508 ± 0.2246	0.0106 ± 0.0092	0.8725 ± 0.1214	0.0494 ± 0.0234
	Hybrid-MDN-NoGate	**0.2285 ± 0.1436**	**0.2857 ± 0.1739**	**0.0070 ± 0.0053**	0.9386 ± 0.0381	0.0425 ± 0.0221
	GH-MDN	0.2302 ± 0.1476	0.2863 ± 0.1759	0.0071 ± 0.0053	0.9830 ± 0.0143	0.0549 ± 0.0264
Conductivity	LSTM-Baseline	8.5661 ± 2.6766	19.9272 ± 5.7090	–	–	–
	LSTM-ConstantVariance	8.8534 ± 2.9071	20.3269 ± 6.3227	0.0207 ± 0.0053	0.9050 ± 0.0702	0.0786 ± 0.0203
	Hybrid-MDN-NoGate	6.5138 ± 1.6176	19.3036 ± 5.5194	**0.0126 ± 0.0037**	0.9647 ± 0.0102	0.0776 ± 0.0151
	GH-MDN	**6.5111 ± 1.5631**	**19.1718 ± 5.3887**	0.0136 ± 0.0040	0.9933 ± 0.0051	0.1313 ± 0.0315
DO	LSTM-Baseline	0.2334 ± 0.0332	0.2954 ± 0.0443	–	–	–
	LSTM-ConstantVariance	0.2346 ± 0.0354	0.2963 ± 0.0459	0.0184 ± 0.0041	0.9332 ± 0.0426	0.1182 ± 0.0177
	Hybrid-MDN-NoGate	0.2250 ± 0.0403	0.2840 ± 0.0518	0.0172 ± 0.0040	0.9450 ± 0.0233	0.1158 ± 0.0201
	GH-MDN	**0.2204 ± 0.0443**	**0.2781 ± 0.0570**	**0.0171 ± 0.0042**	0.9809 ± 0.0154	0.1415 ± 0.0249

**Table 4:** Statistical significance analysis of the improvement in CRPS provided by the GH-MDN architecture compared to the LSTM-ConstantVariance baseline. The best performance (lowest Mean CRPS) is bolded.

Statistical Metric	Result
Mean CRPS (Baseline)	0.0138
Mean CRPS (GH-MDN)	0.0101
Mean Improvement	0.0036
95% Bootstrap Confidence Interval	[0.0032,0.0040]
Wilcoxon Signed-Rank Test (*p*-value)	(p≪0.001)

**Table 5:** Aggregated performance comparison of the final tuned MDN models, highlighting the trade-off between CRPS, sharpness (MPIW), and reliability (PICP).

Target Variable	Model	CRPS (Mean ± Std)	MPIW (Scaled)	Calib. PICP (Target: 0.95)	CRPS Winner
Turbidity (Volatile)	GH-MDN	0.0053 ± 0.0021	0.0288	0.9806	(Tie)
	Hybrid-MDN-NoGate	0.0051 ± 0.0021	0.0160	0.9719	
Conductivity (Volatile)	GH-MDN	0.0136 ± 0.0040	0.1313	0.9933	Hybrid-MDN-NoGate
	Hybrid-MDN-NoGate	0.0126 ± 0.0037	0.0776	0.9647	
pH (Smooth)	GH-MDN	0.0067 ± 0.0019	0.0591	0.9883	Hybrid-MDN-NoGate
	Hybrid-MDN-NoGate	0.0060 ± 0.0014	0.0431	0.9577	
Temp (Smooth)	GH-MDN	0.0071 ± 0.0053	0.0549	0.9830	(Tie)
	Hybrid-MDN-NoGate	0.0070 ± 0.0053	0.0425	0.9386	
DO (Smooth)	GH-MDN	0.0171 ± 0.0042	0.1415	0.9809	GH-MDN
	Hybrid-MDN-NoGate	0.0172 ± 0.0040	0.1158	0.9450	

**Table 6:** Architectural trade-offs in point-forecast accuracy. The best performance (lowest MAE) per variable is highlighted in bold.

Model Configuration	DO	Temp	pH	EC (μS/cm)
GH-MDN (Ours)	1.95	**0.23**	**0.012**	6.51
M2P Guided-Attention	**0.66**	0.55	0.15	**5.17**

**Table 7:** Performance vs. computational cost for the Turbidity target.

Model	CRPS	Mean Training Time (s)	Mean Inference Time (s)
LSTM-Baseline	–	104.45	0.87
LSTM-ConstantVariance	0.0113	117.76	0.86
Hybrid-MDN-NoGate	0.0055	475.20	2.71
GH-MDN	0.0055	539.62	2.88

**Table 8:** CRPS comparison of sequential vs. end-to-end training. The best result is highlighted in bold.

Training Strategy	Mean CRPS (Lower Is Better)
Sequential Training	**0.0053**
End-to-End Training	0.0055

## Data Availability

The specific dataset synthetic_river_wq_hourly_deep_learning.csv can be reproduced using the provided generation script with the default seed. The synthetic data and its generation script are available at https://github.com/Nehmimed/GH_MDN_StackedLSTM (accessed on 10 December 2025).

## Appendix A. Synthetic Data Generation Algorithm

The synthetic WQ time-series data was generated to emulate key characteristics observed in real-world hourly data, including seasonality, trends, noise, inter-parameter correlations, and importantly, event-driven heteroscedasticity for specific parameters like Turbidity and Conductivity. Algorithms A1–A3 outline the conceptual steps involved in this process. The parameters used in the generation, such as amplitudes, means, and storm characteristics, were chosen to produce challenging yet realistic time series for evaluating the forecasting models.

**Algorithm A1** Synthetic data generation: base components
1: **function** GenerateBaseComponents(t,is_storm,intensity,P) 2: ▹ Generates a matrix B of deterministic base signals 3: $T_{seas}\leftarrow P_{T.mean}\;+\;$Seasonal$\left(t,P_{T.seas\_amp},\ldots \right)$ 4: $T_{diel}\leftarrow$ Diel$\left(t,P_{T.diel\_amp},\ldots \right)$ 5: $B_{Temp}\leftarrow T_{seas}+T_{diel}$ 6: $DO_{sat}\leftarrow 14.652-0.41B_{Temp}+0.008B_{Temp}^{2}-0.000078B_{Temp}^{3}$ ▹ DO saturation polynomial 7: $DO_{photo\_amp\_mod}\leftarrow P_{DO.photo\_amp}\cdot (1+0.3\;\cdot$ Seasonal(t,1,…)) 8: $DO_{bio}\leftarrow$Diel$\left(t,DO_{photo\_amp\_mod},P_{DO.peak\_h},P_{DO.trough\_h}\right)$ ▹ Photosynthesis/respiration 9: $B_{DO}\leftarrow \left(DO_{sat}\cdot P_{DO.sat\_factor}\right)+DO_{bio}$ 10: $pH_{seas}\leftarrow P_{pH.mean}\;+\;$Seasonal$\left(t,P_{pH.seas\_amp},\ldots \right)$ 11: $pH_{diel}\leftarrow \left(\frac{DO_{bio}}{E[DO_{photo\_amp\_mod}]+\epsilon }\right)\cdot P_{pH.diel\_amp}\cdot 1.5$ ▹ pH cycle coupled to DO 12: $B_{pH}\leftarrow pH_{seas}+pH_{diel}$ 13: $Cond_{seas}\leftarrow P_{C.mean}\;+\;$Seasonal$\left(t,P_{C.seas\_amp},\ldots \right)$ 14: $Cond_{dilution}\leftarrow \left(P_{C.dilution\_factor}\cdot \mathrm{intensity}\right)⊙Cond_{seas}$▹⊙ is element-wise product 15: $B_{Cond}\leftarrow Cond_{seas}-Cond_{dilution}⊙\mathrm{is}\_\mathrm{storm}$ ▹Apply dilution only during storms 16: $Turb_{spikes}\leftarrow \left(P_{Tu.spike\_factor}\cdot {\mathrm{intensity}}^{1.5}\right)⊙$ Sample(L≀}\≀∇⇕⊣↕(0,0.5,n)) 17: $B_{Turb}\leftarrow P_{Tu.mean}+Turb_{spikes}⊙\mathrm{is}\_\mathrm{storm}$ 18: **return** matrix $B=[B_{Temp},B_{DO},B_{pH},B_{Cond},B_{Turb}]$

**Algorithm A2** Synthetic data generation: correlated residuals
1: **function** GenerateCorrelatedResiduals($n,P_{noise}$) 2: ▹ Generates multivariate AR(1) noise matrix R 3: Initialize $\Sigma_{innov}$ as a diagonal matrix with variances from $P_{noise}$ 4: **for** each correlation $\rho_{ij}$ between variables i,j in $P_{noise}$ **do** 5: $\sigma_{i},\sigma_{j}\leftarrow \sqrt{\Sigma_{innov}\left[i,i\right]},\sqrt{\Sigma_{innov}\left[j,j\right]}$ 6: $\Sigma_{innov}\left[i,j\right]\leftarrow \Sigma_{innov}\left[j,i\right]\leftarrow \rho_{ij}\sigma_{i}\sigma_{j}$ 7: **if** $\Sigma_{innov}$ is not positive definite **then** 8: $\Sigma_{innov}\leftarrow \mathrm{diag}\left(P_{noise.variances}\right)$ ▹ Fallback to no correlation 9: $\mathrm{ffl}∼N(0,\Sigma_{innov})$ for *n* timesteps 10: Initialize residuals matrix R of size n×|vars| 11: R[1,:]←ffl[1,:] 12: **for** t=2 to *n* **do** 13: R[t,:]←Φ⊙R[t−1,:]+ffl[t,:] ▹Φ is the vector of AR(1) coefficients 14: **return** R

**Algorithm A3** Synthetic data generation: final combination
1: **function** CombineAndFinalize(B,R,is_storm,P) 2: $X_{final}\leftarrow B+R$ ▹ Initial combination 3: ▹ Special reconstruction for Turbidity 4: $Turb_{bkg\_noise}\leftarrow$ Sample$(L≀\}\backslash ≀\nabla ⇕⊣↕\left(\log\left(P_{Tu.mean}\cdot 0.2+\epsilon \right),P_{Tu.\sigma \_base},n\right))$ 5: $Turb_{resid\_dampened}\leftarrow R_{Turb}⊙\left(1-0.8\cdot \mathrm{is}\_\mathrm{storm}\right)$ 6: $X_{final.Turb}\leftarrow B_{Turb}+Turb_{bkg\_noise}+Turb_{resid\_dampened}$ 7: $X_{final.Turb}\left[\mathrm{is}\_\mathrm{storm}\right]\leftarrow B_{Turb}\left[\mathrm{is}\_\mathrm{storm}\right]$ ▹ Storm spikes dominate over other noise 8: ▹ Introduce stochastic hypoxia events 9: **for** t=1 to *n* **do** 10: **if** rand$\left(\right)<P_{DO.hypoxia\_prob}$ **then** 11: *h* ← Sample$\left(E\left(\mathrm{scale}=P_{DO.hypoxia\_sev}\right)\right)$ ▹ Sample from Exponential dist. 12: $X_{final.DO}\left[t\right]\leftarrow X_{final.DO}\left[t\right]-h$ 13: ▹ Clip all variables to plausible physical/instrumental limits 14: $X_{final}\leftarrow$ ClipToBounds$\left(X_{final},P_{limits}\right)$ 15: **return** $X_{final}$

This process allows for the creation of datasets where the efficacy of models in handling event-driven heteroscedasticity can be systematically evaluated.
